# HIV Pre-Exposure Prophylaxis (PrEP)—A Quantitative Ethics Appraisal

**DOI:** 10.1371/journal.pone.0022497

**Published:** 2011-08-05

**Authors:** Madzouka B. Kokolo, Dean A. Fergusson, D. William Cameron

**Affiliations:** 1 Ottawa Hospital Research Institute, Clinical Epidemiology Programme, Ottawa, Ontario, Canada; 2 University of Ottawa at The Ottawa Hospital, Ottawa, Ontario, Canada; Umeå University, Sweden

## Abstract

**Background:**

There is now strong evidence that preventive oral antiretroviral therapy can moderately reduce likelihood of HIV infection. This concept is called HIV pre-exposure prophylaxis (PrEP). Premature closures of some previous PrEP clinical trials, secondary to ethical concerns, did not stop research. We aimed to appraise the extent of ethics considerations reporting in PrEP study documents.

**Methods:**

We conducted a systematic quantitative ethics appraisal, grounded in PrEP literature and using eight principles proposed by Ezechiel Emanuel. We developed an *a priori* checklist of 101 evidence-based ethics items. We obtained protocols for eleven of nineteen clinical controlled studies identified. Two reviewers independently appraised study documents against the checklist. Ethics appraisal was synthesized using adjusted percentages of items reported.

**Results:**

On average, 58% of the 101 ethics items were mentioned or addressed in documents, with variations noted both across studies and across principles. Considerations pertaining to *social value* were least reported (43% of checklist items, on average) whereas considerations related to *informed consent* and *favorable risk-benefit ratio* were most reported (75% of checklist items, on average).

**Discussion:**

Some PrEP studies reportedly address more ethics considerations than others but, overall, ethics considerations reporting could be much improved. While this review does not allow us to comment on the actual execution of HIV PrEP trials, it is a reminder that optimism generated by potentially effective interventions should not overshadow the importance of ethics in research design and development. Improving ethics reporting might improve the perceived value of PrEP research and subsequent data.

## Introduction

Pre-exposure prophylaxis (PrEP) is an approach whereby preventive treatment is taken by someone without a certain condition, before exposure to agent(s) causing that condition. HIV PrEP semantically encompasses all preventive approaches aiming at reducing the susceptibility of seronegatives to HIV infection, in anticipation of a high risk exposure. So, *stricto sensu*, all of the following belong to HIV PrEP: behaviors and interventions for the control of sexually transmitted diseases, male/female condom use, male circumcision, experimental prophylactic vaccines, vaginal and rectal experimental microbicides (containing HIV antiretrovirals or not), and experimental systemic administration of HIV antiretrovirals (oral or parenteral).

The trend has been to associate PrEP to microbicides but also, and more often, to oral antiretrovirals (ARVs) for primary HIV prevention. For our study, we opted to follow the definition of some influential stakeholders who reserve the acronym PrEP to the latter (e.g., www.cdc.gov/hiv/prep, www.avac.org). Among key differences between microbicides and oral ARVs, antiretrovirals tested for prevention are already available on the market. This means, among other ethical challenges, that oral ARVs for PrEP can be provided “off-label” to seronegatives by professionals or patients with access to these drugs. Indeed, some people may choose, for whatever reason, not to wait for official endorsement of PrEP regimen by competent public health authorities.

The oral PrEP concept has been applied to HIV through experimental clinical use of therapeutic antiretrovirals for about a decade [1]. Because oral antiretrovirals currently tested in humans are already marketed, PrEP could become the next available new technology for HIV biomedical prevention. The proof-of-concept has recently been demonstrated for oral daily emtricitabine/tenofovir in men having sex with men, with a 44% reduction in seroconversion risk [2]. Although these results are encouraging, this level of efficacy was deemed insufficient to justify large scale implementation.

Going back just a few years, HIV PrEP research faced serious challenges. In 2004–2005, three PrEP trials were prematurely closed (Cambodia [3], Malawi [4], West Africa [5]). In all cases, decision to close study sites was not based on data analyses but followed ethical concerns publicly voiced by community advocates [6]–[8]. Also, investigators of two effectiveness studies (in Botswana) experienced methodological dilemmas in trial execution. These issues resulted in premature termination to allow for a switch of test-drug (in 2007), for one trial [9], and to the inability to assess effectiveness, for the other (in 2009) [10].

Following the “community-triggered” early closures, international stakeholders were consulted to determine what had gone wrong [11]. Methodological and ethics guidelines specific to biomedical HIV prevention research ensued [12]–[14]. Although those guidelines were developed to positively influence future PrEP research conduct, it is unclear whether they have been taken up by PrEP investigators, regulators and sponsors. Meanwhile, the number of HIV PrEP clinical trials has been growing [15], as has HIV PrEP funding, with a cumulative 173 million dollars invested, between 2002 and 2009 [16].

Our study aimed to 1) appraise the extent to which ethics considerations are reported in HIV oral PrEP study documents and 2) identify the least and the most reported ethics considerations.

## Methods

### Identification of eligible studies and acquisition of source documents

A systematic search of multiple information sources identified prospective clinical trials designed to test approved systemic antiretrovirals for HIV PrEP, in humans [15]. We obtained trials' descriptions from trial registries (www.clinicaltrials.gov). Unless full protocols and consent forms were found online (www.mtnstopshiv.org), we communicated directly with investigators or sponsors to request last approved versions. Published trial reports were also retrieved (Medline, Embase).

### Ethics analysis framework

We, the three authors of this paper, developed a checklist *a priori*, in a stepwise process. MBK was then a graduate student who had previously written an essay on the ethics of the PrEP trial stopped in Cameroon (http://mk-publications-en.yolasite.com/resources/Kokolo2005_unpublished.pdf). DAF is a methodologist and senior scientist with expertise in clinical trials design and bioethics (www.ohri.ca/profiles/fergusson.asp). DWC is a medical doctor, an infectious diseases specialist and a senior scientist with expertise in trials involving resource-limited countries (www.ohri.ca/profiles/cameron.asp).

First, we identified guidance documents of reference. We considered the 19 guidance points proposed as *Ethical Considerations in Biomedical HIV Prevention Trials* by UNAIDS and WHO [13]. We also retained the Institute Of Medicine's 43 recommendations on *Methodological Challenges in Biomedical HIV Prevention Trials* [12]. For simplicity and for synthesis purposes, we used Emanuel et al.'s *Ethical Principles and Benchmarks for Multinational Clinical Research* [17] as our overall framework. This model features 31 benchmarks classified in eight principles: *collaborative partnership*, *social value*, *scientific validity*, *fair selection of study population*, *favorable risk-benefit ratio*, *independent review*, *informed consent*, and *respect for recruited participants and study community*. Additionally, we identified reported ethical issues about HIV PrEP, through a screening of peer-reviewed articles published until 2008.

Second, we made an initial list of items that would make up our checklist. Our intent was to have an inventory of conditions that should be fulfilled or at least discussed, in accordance with the ethics guidance documents we retained. While going through each guidance document, we extracted concepts of ethical considerations deemed relevant to HIV oral PrEP. Key papers from our literature review provided a few more items. We reviewed the checklist and discussed its comprehensiveness.

Third, we standardized the formulation of our items. Each item was an affirmative stand-alone statement representing a practical ethical consideration. Because our ethics guidance sources presented many redundant considerations within and across documents, we removed repetitive statements. After three rounds of discussion, we reached a consensus to retain 101 checklist items.

Fourth, we structured our checklist by domains. Each checklist item was categorized as belonging to one of the 31 benchmarks, hence building a second level of specification for the 8 principles, as suggested by Emanuel et al. [17]. We numbered checklist items, for easier referencing. Clarifications and references to original ethics guidelines were inserted as endnotes to facilitate interpretation and harmonize appraisal by independent reviewers. For instance, under *Community Participation* principle, item number 12 - “*standard ethics training given to research staff in all study sites*” - was drawn from a sentence in UNAIDS/WHO's guidance document: “*Research literacy programs that include ethics training for study staff can facilitate and enhance cooperation with civil society groups*.” (Guidance point 2: Community Participation). And under *Scientific Validity* principle, item number 31 - “*description of strategies for achieving accrual rate goals and for maximizing retention*” - was derived from an IOM's recommendation stating that “…*investigators should place a high priority on developing effective strategies to achieve accrual rate goals and to minimize losses to follow-up*.” (Chapter 6-Design Considerations: Recruitment and Retention).

### 101 data items for eight ethics principles

*Collaborative partnership* (6 benchmarks) has 20 items; *social value* (4 benchmarks) has 7; *scientific validity* (3 benchmarks) has 33; *fair selection of study population* (3 benchmarks) has 6; *favourable risk-benefit ratio* (2 benchmarks) has 4; *independent review* (3 benchmarks) has 10; *informed consent* (5 benchmarks) has 12; and *respect for recruited participants and study community* (5 benchmarks) has 9.

### Data extraction

The ethics checklist was piloted on two included studies and amended to optimize extraction process. Each study document obtained was fully reviewed by two independent assessors, who were specifically trained for data extraction. Our intent was to assess the reporting of ethics considerations in PrEP study documents. So, investigators were not surveyed; neither were they queried on data items found or not found in documents. In case of inconsistency across source documents pertaining to the same study, study report prevailed on consent form, which prevailed on protocol, which prevailed on registry file.

For each study, a given item was checked if 1) corresponding information was clearly identified in a source document and 2) nothing in the rest of same document contradicted that information. Discordant assessments were resolved consensually or with assistance of a third assessor.

### Statistics

Disaggregated counts of items checked were electronically captured and analyzed in Excel® (Microsoft, Inc., version 2003) and in SAS® (version 9.1: SAS Institute Inc., Cary, NC, United State of America). We counted the number of studies (n) for which we found each checklist item. In addition, we calculated the percentage of items checked, for each individual study, at principle level. The denominator used was smaller than the total number of items whenever there were considerations not applicable to a study (e.g., pregnancy-related issues in men only trial). We also calculated the percentage of items checked, for each study, across principles. Because the number of items varied under each principle, we adjusted these proportions through a direct standardization [18], assuming all principles had the same weight [19].

Median percentages of ethics items reported were calculated, across studies and across principles (rounded up when first decimal equalled 5). Ethical principles for which the ethics considerations were least reported or most reported were identified using those medians. Sub-group analyses were also based on a comparison of median percentages computed. We chose *a posteriori* to assess influence of studies' primary question (efficacy/effectiveness versus other primary outcomes), sites location (enrolment involving populations in high income countries versus enrolment not involving them), and progress status (studies with early closure of one or all study sites versus studies ongoing or completed as planned). Our literature review had suggested that such factors could impact trials' acceptability, which has strong links with ethics. For progress status, we further compared median percentages of ethics items reported between studies closed early on the initiative of investigators (e.g., a priori defined futility) and studies closed early following community pressure. Analysis was restricted to eligible studies for which at least an approved protocol could be obtained.

## Results

Out of 19 HIV oral PrEP trials identified, we excluded one that focused on topical PrEP (compared to oral tenofovir taken only once) [20], [21] and three that were still in protocol development phase at the time we completed our data collection [22]–[24]. We also excluded four studies for which only one target document (registry file or report) was obtained [1], [4], [25], [26]. In those cases, contacted investigators did not respond (n = 1), declined to release documents (n = 2) or explained that protocol had never been approved (n = 1). All four target study documents (registry file [27]–[29], protocol [30]–[32], consent form [33]–[35], report [2], [3], [5]) were obtained for three studies, and three (registry file [36]–[43], protocol [44]–[51], consent form [45]–[49], [51]–[53]) were obtained for eight studies. Those 11 studies were included in our ethics analysis (Tables 1 and 2).

**Table 1:** Characteristics of the 11 PrEP trials appraised.

Lead investigator/Registration code	Sponsors	Enrolled	Population	Countries
Celum/NCT00557245	BMGF, Gilead, UW	(4700)	serodiscordant couples (hetero)	Kenya, Uganda
Chirenje/NCT00705679	CONRAD, Gilead, NIH	(4200)	females (hetero)	Malawi, South Africa, Uganda, Zimbabwe
Choopanya/NCT00119106	CDC, Gilead	(2400)	females+males injecting recreational drugs (unspecified sexual orientation)	Thailand
Grant/NCT00458393	BMGF, Gilead, NIH	2499	males (homo)	Brazil, Ecuador, Peru, South Africa, Thailand, USA
Grohskopf/NCT00131677	CDC, Gilead	400	males (homo)	USA
Hendrix/NCT00592124	CONRAD, Gilead, NIH	144	females (hetero)	South Africa, Uganda, USA
Page-Shafer/NCT00078182	BMGF, FHI, Gilead, NIH	0	females (hetero)	Cambodia
Peterson/NCT00122486	BMGF, FHI, Gilead, NIH	936	females (hetero)	Cameroon, Ghana, Nigeria
Smith/NCT00111150	CDC, Gilead	71	females+males (hetero)	Botswana
Thigpen/NCT00448669	CDC, Gilead	(1200)	females+males (hetero)	Botswana
Van Damme/NCT00625404	BMGF, FHI, Gilead, USAID	(3900)	females (hetero)	Kenya, Malawi, South Africa, Tanzania

Legend:

Registration code: as per the National Institute of Health trials registry.

Sponsors: BMGF = Bill and Melinda Gates Foundation; CDC = Centers for Disease Control and Prevention (United States of America); Gilead = Gilead Sciences Incorporation; CONRAD = University of Eastern Virginia's CONtraceptive Research & Development program; FHI = Family Health International; NIH = National Institutes of Health (United States of America); USAID = United States Aid for International Development; UW = University of Washington.

Enrolled: number in brackets represent target sample sizes; other numbers are actual sample sizes reported.

Population: homo = homosexual; hetero = heterosexual.

Countries: USA = United States of America.

**Table 2:** Design and progress status of the 11 PrEP trials appraised.

Lead investigator/Registration code	Pill(s) tested	Comparator(s)	Primary outcome(s)	Timeline (activation to closure)	Progress status
Celum/NCT00557245	1) FTC/TDF; 2) TDF	matched placebo	efficacy, safety	2008-	ongoing
Chirenje/NCT00705679	1) FTC/TDF; 2) TDF	1) TDF gel; 2) matched placebo pill; 3) matched placebo gel;	effectiveness, extended safety	2009-	ongoing
Choopanya/NCT00119106	TDF	matched placebo	efficacy, safety	2005-	ongoing
Grant/NCT00458393	FTC/TDF	matched placebo	efficacy, safety	2007–2010	completed
Grohskopf/NCT00131677	TDF	1) delayed TDF; 2) matched placebo; 3) delayed matched placebo	extended safety, tolerability	2005–2010	completed
Hendrix NCT00592124	TDF	1) TDF gel; 2) “self” (cross-over design)	adherence, acceptability, pharmacokinetics	2008–2010	completed
Page-Shafer/NCT00078182	TDF	matched placebo	safety, efficacy	2004	closed early (community)
Peterson/NCT00122486	TDF	matched placebo	effectiveness, extended safety	2004–2006	closed early (community)
Smith/NCT00111150	TDF	matched placebo	extended safety, efficacy	2005–2007	closed early (investigators∶upgrade to Thigpen's trial)
Thigpen/NCT00448669	FTC/TDF	matched placebo	extended safety, efficacy	2007–2009	closed early (investigators∶futility)
Van Damme/NCT00625404	FTC/TDF	matched placebo	effectiveness, safety	2009–2011	closed early (investigators∶futility)

Legend:

Registration code: as per the National Institute of Health trials registry.

Pill(s) tested: FTC = emtricitabine; TDF = tenofovir.

### PrEP trials analyzed

The 11 trials analyzed were first registered between 19 February 2004 and 24 June 2008, and were scheduled to start between June 2004 and June 2009. At the time we completed our analysis, 3 trials were completed, 5 had closed prematurely, and 3 were ongoing. Nine trials analyzed had efficacy/effectiveness as a primary outcome, while the other two had only safety/tolerability, adherence, acceptability, and/or pharmacokinetics as primary outcomes. Eight trials were to recruit in low or middle income countries only, while 3 were to include sites in the United States of America (USA). Trials were designed to test daily tenofovir and/or oral emtricitabine oral pills versus placebo or other comparator. Target populations included adult heterosexual females, men having sex with men, stable serodiscordant heterosexual couples, and users of injectable recreational drugs. All trials were sponsored by institutions based in the USA. The 11 approved protocols were dated between 13 August 2004 and 29 May 2008. Five protocols were versions 1.x, one was a version 2.0, three were version 3.x, one was a version 4.0, and one was a version 7.0. Those protocols were collected between October 2008 and January 2009 (Tables 1 and 2).

### Ethics appraisal

#### All trials combined

**Figure 1:**
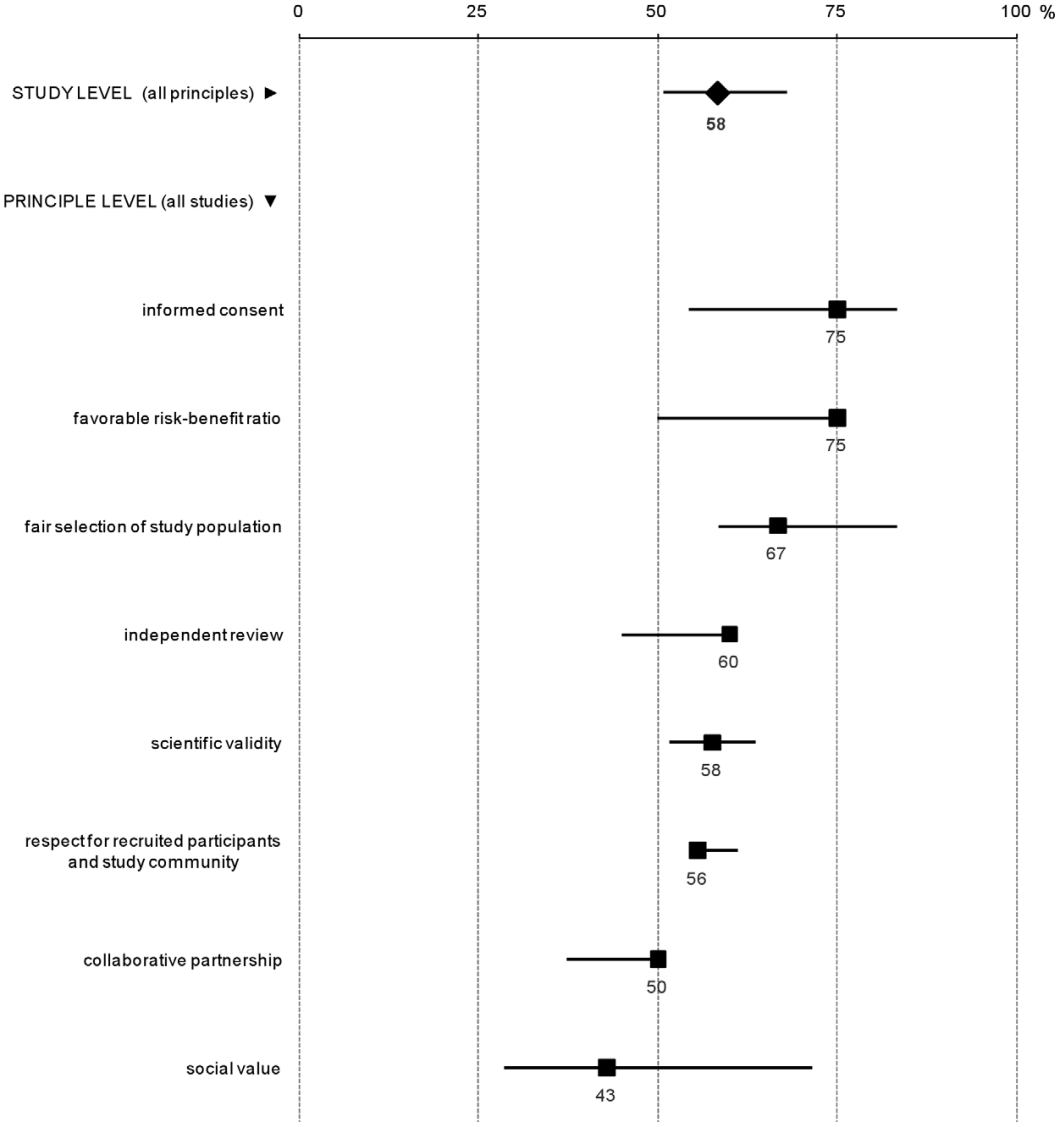
Average percentages of ethics items reported, all studies combined. Legend: The first line represents the interquartile range of standardized percentages of ethics items reported in PrEP trial documents, out of a list of 101 items, with the median percentage represented by a diamond (all principles combined). The other 8 lines represent interquartile ranges of the percentages of items reported for each ethics principle listed, with each median percentage represented by a square. The denominator used varied with ethics principles: 20 for *collaborative partnership*; 7 for *social value*; 33 for *scientific validity*; 6 for *fair selection of study population*; 4 for *favourable risk-benefit ratio*; 10 for *independent review*; 12 for *informed consent; and* 9 for *respect for recruited participants and study community*.

**Figure 2:**
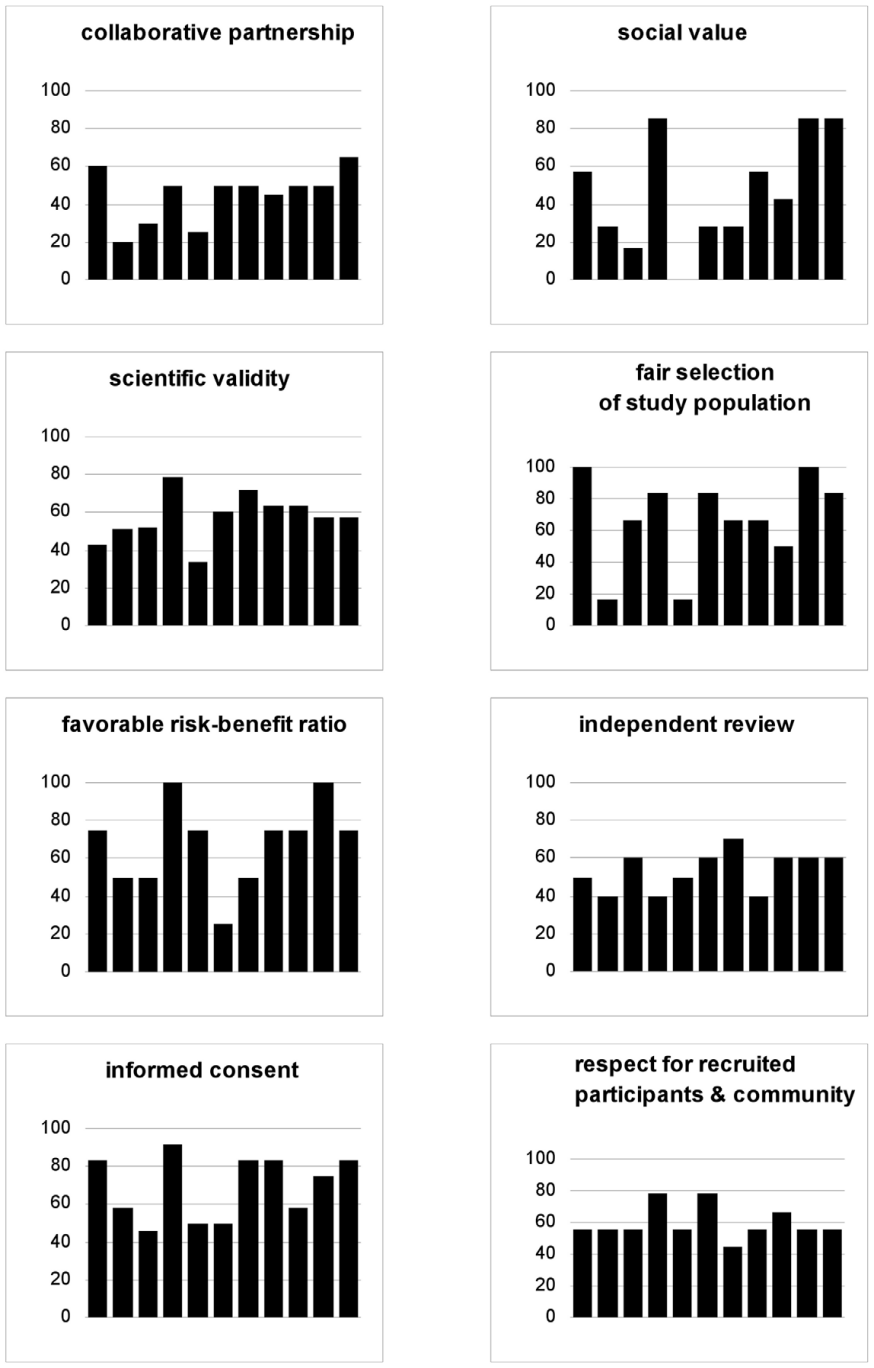
Percentage of ethics items reported: individual studies, by principle. Legend: each bar represents adjusted percentage of ethics items documented for a given trial; bars are ordered from earliest to most recent trial, based on source documents' date (not based on study activation date).

Out of 101 checklist items, 14 were found for all studies analyzed (as much as items were applicable); 43 were found for half studies or less; and six were found for no study: *strategy to ensure legitimacy of community partners chosen to represent host community, randomized comparisons of behavioral risk-reduction interventions incorporated into design, behavioral co-intervention was field tested during planning phase, specification of measures taken to ensure independence and competence of ethics review, specification of measures taken to prevent situations of conflict(s) of interest, and strategy to assist local ethics committee in reaching international standard procedures* (Table S1). At study level, adjusted percentages of ethics items reported, all principles combined, were between 38% and 76% (median = 58%; interquartile range = 51–68%). At principle level, median percentages of ethics items reported, all studies combined, were between 43% (*social value*) and 75% (*informed consent* and *favorable risk-benefit ratio*) (Figure 1). Variations were noted in the percentage of ethics items reported by principle, between individual studies, especially for *social value* (Figure 2).

#### Efficacy/effectiveness trials (n = 9) versus non-efficacy/effectiveness trials (n = 2)

All median proportions of ethics items reported for efficacy studies were equal to or higher than corresponding values for studies not having efficacy/effectiveness as a primary outcome. At study level, median percentage of ethics items reported, all principles combined, was 61% (18% more than for studies with other primary focus). At principle level, median percentages of ethics items reported, all efficacy/effectiveness studies combined, were between 50% (*collaborative partnership*) and 83% (*fair selection of study population* and *informed consent*) (Table 3).

**Table 3:** Median percentages of ethics items reported in PrEP study documents: sub-group ethics appraisal.

	All principles combined	Collaborative partnership	Social value	Scientific validity	Fair sselection of study population	Favorable risk-benefit ratio	Independent review	Informed consent	Respect for recruited participants & study community
**Primary outcome**									
**efficacy (n = 9)**	**61**	**50**	**57**	**61**	**83**	**75**	**60**	**83**	**56**
other outcome(s) (n = 2)	43	30	8	43	42	63	55	48	56
**Enrollment sites**									
**no USA site (n = 8)**	**63**	**50**	**57**	**59**	**83**	**75**	**55**	**79**	**56**
at least 1 USA site (n = 3)	48	35	17	52	67	50	60	50	56
**Progress status**									
**early closure (n = 5)**	**65**	**50**	**57**	**58**	**83**	**75**	**50**	**83**	**56**
**2 community-triggered early closures**	**53**	**40**	**43**	**47**	**58**	**63**	**45**	**71**	**56**
**3 investigators-triggered early closures**	**71**	**50**	**86**	**58**	**83**	**75**	**60**	**83**	**56**
no early closure (n = 6)	56	50	29	62	67	63	60	54	61

#### Trials without USA site (n = 3) versus trials with USA site(s) (n = 8)

Most median proportions of ethics items reported for studies without USA site were equal to or higher than corresponding values for studies with USA site(s). *Independent review* was the exception (5% fewer items reported for trials without USA site). At study level, median percentage of ethics items reported, all principles combined, was 63% (15% more than for studies with USA site-s). At principle level, median percentages of ethics items reported, all studies without USA site combined, were between 50% (*collaborative partnership*) and 83% (*fair selection of study population*) (Table 3).

#### Trials fully or partially closed early (n = 5) versus trials not closed early (n = 6)

Most median proportions of ethics items reported for studies closed early were higher than or equal to corresponding values for studies not closed early. *Scientific validity*, *independent review* and *respect of recruited participants and study community* were exceptions (4%, 10%, and 5% fewer items reported for trials closed early, respectively). At study level, median percentage of ethics items reported, all principles combined, was 65% (9% more than for studies not closed early). At principle level, median percentages of ethics items reported, all early closures combined, were between 50% (*collaborative partnership* and *independent review*) and 83% (*fair selection of study population* and *informed consent*). Studies closed early following community pressure had fewer items reported, compared to studies closed early based on stopping rules defined *a priori*, except for *respect of recruited participants and study community* (equal percentage) (Table 3).

### Ethics guidelines reported

Ten protocol teams (91%) cited ethics guidance documents. Nine of those mentioned international guidelines (Declaration of Helsinki [54] or International Conference on Harmonisation-Guidelines for Good Clinical Practice [55]) and/or guidelines developed in sponsoring country (e.g., USA Code of Federal Regulations, standard operating procedures). One team cited UNAIDS/AVAC's Good Participatory Guidelines [14].

## Discussion

Based on our analytic framework, 58% of 101 ethics items relevant to HIV PrEP were found in study documents, on average. As many as 43 of those items were reported for half studies analyzed or less. We demonstrated variation in reporting across the 11 studies appraised and across the eight ethical principles explored. Our quantitative analytic strategy allowed us to minimize assessment subjectivity. And Table S1 displays data detailed enough to permit the reader to weigh the importance of each item, based on his or her own perspective. Rather than singling out individual trials (e.g., in Figure 2), we chose to present results as aggregate figures so as to evaluate HIV PrEP trials as a field, and hopefully generate constructive discussions that may guide positive adjustments. Indeed, our data suggest that ethics considerations reporting can be much improved, overall.

At study level, more ethics considerations were reported for efficacy/effectiveness trials than for trials focusing on other primary questions (i.e., safety, tolerability, adherence, acceptability, pharmacokinetics) –18% more checklist items, on average. Sub-group analysis based on this factor also showed the largest differences, in all but three principles. This is encouraging, considering that efficacy trials are largest in sample sizes and that their results are always highly anticipated for public health decision-making [56]. However, as per the general trend, items related to *collaborative partnership*, *respect for recruited participants and study community*, and *social value* were the lowest reported in this subset (50%, 56% and 57%, respectively). This seems dissonant with the repeated argument that PrEP could be most beneficial to populations living in areas of highest HIV prevalence [57]–[59]. More discussion is required regarding roll-out capacities and planning.

Similarly, more ethics considerations were reported for studies conducted outside of the USA, compared to studies with at least one USA site (15% more checklist items, on average). Sub-group analysis based on this factor showed the largest differences for *favorable risk-benefit ratio* (25% more items, for trials outside the USA). In our sample, all eight studies without USA sites have been led, co-led and/or sponsored by USA-based institutions [15]. Other host communities were in low-and-middle income countries (South-America, Sub-Saharan Africa, South-East Asia). Our findings might reflect institutional limitations in clinical research regulations, in those countries [60]–[62]. Multiple-level regulatory reviews imposed on investigators may also influence reporting of ethics-relevant matters in international trials.

Also, we found no correlation between study status and percentage of ethics item reported: some ongoing/completed trials had fewer ethics considerations reported than some trials halted earlier (data not presented). This was unexpected since ethics and methods concerns were clearly reported as leading reasons for premature site closures [63]. At principle level, studies closed early had fewer ethics items reported for *scientific validity* (58%), *independent review* (50%) and *respect for recruited participants and study community* (56%), compared with studies not closed prematurely (62%, 60% and 61%, respectively). Reporting of ethics considerations was almost always lower for trials stopped following community pressure, compared to trials closed early based on *a priori* stopping rules. This suggest that 1) reporting ethics considerations in protocols may not guarantee that studies will be *perceived* as ethical in host community context; 2) gaps in *scientific validity*, *independent review*, and *respect for recruited participants and study community* may have been core issues in halted trials (although not necessarily the cause of termination); 3) there is a need to further promote and enforce ethics guidelines that were developed for biomedical HIV prevention trials, and from which most of our checklist items were derived [12]–[14].

The validity of our results should be considered in light of some limitations. First, although our checklist was evidence-based, it was developed without extensive experts' consultation or external validation [64]. Second, the analysis of a wider range of study documents (e.g., standard operating procedures, clinical trial agreements/contracts) could have allowed us to find more ethics items reported. Besides, ethics guidance documents we used as references were published after some studies were terminated; however, the systematic nature of our appraisal provided a framework common to all studies and facilitated synthesis. Also, we chose to be conservative in equally weighing all ethics items -as per Emanuel's suggestion [19] - although some may have been given more importance depending on perspective and context. Additionally, documents obtained were at different stages of revision, which may have affected comparability across studies. Most importantly, we did not evaluate the actual conduct of HIV oral PrEP trials, only the extent to which ethics considerations are reportedly addressed in selected documents. Moreover, we did not consider larger, contextual or consequential ethics issues of applying experimental evidence in settings other than communities that hosted a trial (e.g., treatment accessibility, affordability, sustainability). Finally, we only analyzed a sub-set of eligible studies, despite methodical attempts to obtain all protocols.

### Conclusion

Confidence in science requires trust in scientists, and transparency is needed to cultivate that at every stage of the research process. It is commendable that some PrEP research sponsors now make full-text protocols freely available while it was out of the question, not so long ago. As the lead of the first PrEP trial that got stopped following community pressure, Dr. Page-Shafer reported: “*When concerns about the trial were first raised publicly, it became clear to us that aspects of the trial plans were being portrayed inaccurately. At this point, we proposed to our funding agencies that the then current protocol be posted on our institutional websites, as a means of publicly presenting the trial status and planning, but this proposal was rejected*.” [3]. We believe that openly available study protocols should be the norm [65], and checklists like ours could facilitate ethics appraisal or serve as scales to improve reporting quality of ethics considerations.

Harm prevention in clinical research requires harmonized views on ethics principles between funders and hosts of PrEP research projects, and functional regulatory mechanisms in both sponsoring and hosting countries. Proclaimed ethics considerations should be more clearly and more extensively addressed in protocols, so as to alleviate legitimate concerns regarding actual trial conduct. And we showed that there is room for improvement in that area. Sustained vigilance is essential to keep ethics in the foreground throughout the whole research process.

## Supporting Information

Table S1 **Number of study teams reporting each ethics checklist item.** In this table, the 8 principles are guidance terms or expressions representing best practices “that should underlie the conduct of biomedical and behavioral research involving human subjects” (US National commission for the Protection of Human Subjects of Biomedical and Behavioural Research. The Belmont Report: Ethical Principles and Guidelines for the Protection of Human Subjects of Research. 1979). The 31 benchmarks are specific and practical considerations that are “to guide researchers and research-ethics committees in assessing how well the enumerated ethical principles have been fulfilled in particular cases”. The 8 principles and 31 benchmarks presented here were proposed in 2004 (Emanuel EJ, Wendler D, Killen J, Grady C. What makes clinical research in developing countries ethical? The benchmarks of ethical research. J Infect Dis 2004; 189(5):930). The 101 ethics checklist items were formulated by the authors of the present article (MBK, DAF, DWC). n = number of trials reporting checklist item. N = number of trials for which checklist item is relevant. * item irrelevant for the 1 trial designed to be conducted in the USA only. ** item irrelevant for the 2 trials not designed to focus on efficacy/effectiveness. *** item irrelevant for the 2 trials designed to include only men.
(DOC)
