# Supplementary material for: HIV Pre-Exposure Prophylaxis (PrEP)—A Quantitative Ethics Appraisal
Source: PLoS One. 2011 Aug 5;6(8):e22497. doi: 10.1371/journal.pone.0022497 (PMC3151244; doi:10.1371/journal.pone.0022497)
Supplement: Table S1 — Number of study teams reporting each ethics checklist item. In this table, the 8 principles are guidance terms or expressions representing best practices “that should underlie the conduct of biomedical and behavioral research involving human subjects” (US National commission for the Protection of Human Subjects of Biomedical and Behavioural Research. The Belmont Report: Ethical Principles and Guidelines for the Protection of Human Subjects of Research. 1979). The 31 benchmarks are specific and practical considerations that are “to guide researchers and research-ethics committees in assessing how well the enumerated ethical principles have been fulfilled in particular cases”. The 8 principles and 31 benchmarks presented here were proposed in 2004 (Emanuel EJ, Wendler D, Killen J, Grady C. What makes clinical research in developing countries ethical? The benchmarks of ethical research. J Infect Dis 2004; 189(5):930). The 101 ethics checklist items were formulated by the authors of the present article (MBK, DAF, DWC). n = number of trials reporting checklist item. N = number of trials for which checklist item is relevant. * item irrelevant for the 1 trial designed to be conducted in the USA only. ** item irrelevant for the 2 trials not designed to focus on efficacy/effectiveness. *** item irrelevant for the 2 trials designed to include only men. (DOC) [file pone.0022497.s001.doc]

Table S1. Number of study teams reporting each ethics checklist item.

| **Principle** | **Benchmark** | **Item #** |  | **Ethics checklist item** | **n** | **N** |
| --- | --- | --- | --- | --- | --- | --- |
| 1.collaborative partnership | 1.1.develop partnerships with researchers, makers of health policies, and the community | 1 |  | strategy to ensure legitimacy of community partners chosen to represent host community | 0 | 11 |
|  |  | 2 |  | mention of partnership with clinicians/scientist(s) and with policy-makers in host country | 9 | 11 |
|  |  | 3 |  | mention of creative and flexible partnership with HIV and/or non-HIV networks | 8 | 11 |
|  |  | 4 |  | mention of strategy for combining safety information from concurrent trials of similar products | 4 | 11 |
|  | 1.2.involve partners in sharing responsibilities for determining the importance of health problem, assessing the value of research, planning, conducting, and overseeing research, and integrating research into the health-care system | 5 |  | community network/partners for the study are namely identified and their roles clearly defined | 8 | 11 |
|  |  | 6 |  | active contribution of host community in the development of the study question | 3 | 11 |
|  |  | 7 |  | active contribution of local partners in trial planning | 10 | 11 |
|  |  | 8 |  | active contribution of local partners in trial conduct and monitoring | 11 | 11 |
|  |  | 9 |  | mention of a Community Advisory Mechanism (CAM) | 9 | 11 |
|  |  | 10 |  | consultation with community/stakeholders regarding sustainability of interventions locally | 2 | 11 |
|  |  | 11 | ***** | consultation with community/stakeholders regarding sustainability of site, after trial completion | 4 | 10 |
|  | 1.3.respect the community’s values, culture, traditions, and social practices | 12 |  | standard ethics training given to research staff in all study sites | 2 | 11 |
|  |  | 13 |  | intervention(s) deemed culturally appropriate for use among host community | 1 | 11 |
|  |  | 14 |  | strategies to minimize/reduce stigma/discrimination attached to target population | 6 | 11 |
|  | 1.4.develop the capacity for researchers, makers of health policies, and the community to become full and equal partners in the research enterprise | 15 | ***** | mention of a research literacy program for host community | 3 | 10 |
|  |  | 16 | ***** | mention of investment in host country human capacity and physical infrastructure | 3 | 10 |
|  | 1.5.ensure that recruited participants and communities receive benefits from the conduct and results of research | 17 |  | benefits for study participants/host community are clearly delineated | 9 | 11 |
|  | 1.6.share fairly financial and other rewards of the research | 18 | ***** | intellectual property is shared with investigator(s) based in host country | 5 | 10 |
|  |  | 19 |  | some/all data to be owned/co-owned by local institution/organization | 1 | 11 |
|  |  | 20 |  | mention of other agreed sharing of reward | 1 | 11 |
| 2.social value | 2.1.specify the beneficiaries of the research—who | 21 |  | identification of the beneficiaries of the research | 9 | 11 |
|  | 2.2.assess the importance of the health problems being investigated and the prospective value of the research for each of the beneficiaries—what | 22 |  | documented assessment of HIV burden in host community | 7 | 11 |
|  |  | 23 |  | description of social context in host community, as relevant to study conduct | 4 | 11 |
|  | 2.3.enhance the value of the research for each of the beneficiaries through dissemination of knowledge, product development, long-term research collaboration, and/or health system improvements | 24 |  | description of knowledge dissemination plan within host community | 4 | 11 |
|  |  | 25 |  | mention of long-term partnership plan with stakeholder(s) based in host community | 1 | 11 |
|  |  | 26 | ****** | drug proven safe and effective to be made available and affordable to host community, post trial | 8 | 9 |
|  | 2.4.prevent supplanting the extant health system infrastructure and services | 27 |  | discussion on expected impact of trial on health-care system/services in host community | 3 | 11 |
| 3.scientific validity | 3.1.ensure that the scientific design of the research realizes social value for the primary beneficiaries of the research | 28 |  | trial design is informed by social and political study(ies) of host community | 4 | 11 |
|  | 3.2.ensure that the scientific design realizes the scientific objectives while guaranteeing research participants the health-care interventions to which they are entitled | 29 |  | references from previous research/scientific arguments justifying conduct of the study | 11 | 11 |
|  |  | 30 | ****** | use of triangulation for estimation of HIV incidence rate (sample size determination) | 5 | 9 |
|  |  | 31 |  | description of strategies for achieving accrual rate goals and for maximizing retention | 10 | 11 |
|  |  | 32 |  | plans for evaluation of effectiveness of recruitment plan | 9 | 11 |
|  |  | 33 |  | scientific justification of test-drug(s) choice and intervention regimen(s) | 7 | 11 |
|  |  | 34 |  | statement justifying chosen comparator(s) | 6 | 11 |
|  |  | 35 |  | use of both blinded and unblinded control groups | 1 | 11 |
|  |  | 36 |  | randomized comparisons of behavioral risk-reduction interventions incorporated into design | 0 | 11 |
|  |  | 37 |  | quality control measures in the promotion -by research staff- of HIV prevention for participants | 6 | 11 |
|  |  | 38 |  | behavioral co-intervention was field tested during planning phase | 0 | 11 |
|  |  | 39 |  | scientific justification of study timeline | 3 | 11 |
|  |  | 40 |  | strategy to monitor actual degree of exposure of selected participants during trial | 11 | 11 |
|  |  | 41 |  | description of monitoring plan for adherence | 11 | 11 |
|  |  | 42 |  | safety outcome measures include measures for psychological and/or social harm | 10 | 11 |
|  |  | 43 |  | mention of a Data and Safety Monitoring Board (DSMB)/Data Monitoring Committee (DMC) | 10 | 11 |
|  |  | 44 |  | at least one-third of DSMB/DMC members are host country community members | 1 | 11 |
|  |  | 45 |  | clear statement that DSMB/DMC has the option of unblinding to insure participants’ protection | 6 | 11 |
|  |  | 46 |  | statement of basis and criteria for recommendation by DSMB/DMC to modify trial’s size/duration | 4 | 11 |
|  |  | 47 |  | futility stopping rule relies on evidence of a sustained impact on cumulative HIV incidence | 5 | 11 |
|  |  | 48 |  | study will allow provision of information on both short and long-term benefits of intervention | 8 | 11 |
|  |  | 49 |  | HIV testing and treatment plan for potential participants’/participants’ partners | 3 | 11 |
|  |  | 50 |  | each HIV test is to be consented to | 4 | 11 |
|  |  | 51 |  | each HIV testing test is to include pre-test and post-test counseling | 11 | 11 |
|  |  | 52 |  | description of care plan for potential participants screened HIV positive | 5 | 11 |
|  |  | 53 |  | description of care plan for HIV seroconverting participants | 11 | 11 |
|  |  | 54 | ******* | description of complete action plan for participants who become pregnant | 2 | 9 |
|  |  | 55 | ****** | “events-driven” approach for the statistical analysis (actual seroconversion cases) | 6 | 9 |
|  |  | 56 | ****** | primary analytic strategy is intention-to-treat for efficacy endpoint | 9 | 9 |
|  |  | 57 |  | potential impact of adherence is taken into account for statistical analyses | 10 | 11 |
| 3.scientific validity | 3.3.ensure that the research study is feasible within the social, political, and cultural context or with sustainable improvements in the local health-care and physical infrastructure | 58 |  | mention/description of extensive pretrial research in host community | 8 | 11 |
|  |  | 59 |  | mention of involvement of behavioral and social scientists in early planning stages | 2 | 11 |
|  |  | 60 | ***** | consultations/strategies to ascertain technical and material feasibility of trial | 6 | 10 |
| 4. fair selection of study population | 4.1.select the study population to ensure scientific validity of the research | 61 |  | scientific justification of study population, in relation to internal validity | 9 | 11 |
|  |  | 62 |  | scientific justification of study population, in relation to generalizability of results | 5 | 11 |
|  | 4.2.select the study population to minimize the risks of the research and enhance other principles, especially collaborative partnership and social value | 63 |  | justification of study population, based on risk minimization | 8 | 11 |
|  |  | 64 |  | description of educational initiatives to inform host community –at large- about the study | 10 | 11 |
|  | 4.3.identify and protect vulnerable populations | 65 |  | acknowledgment that study population is vulnerable | 4 | 11 |
|  |  | 66 |  | description of measures to minimize risk of exploitation of participants | 8 | 11 |
| 5. favorable risk-benefit ratio | 5.1.assess the potential risks and benefits of the research to the study population in the context of its health risks | 67 |  | HIV strain(s) targeted by study is/are an important public health problem in host community | 2 | 11 |
|  |  | 68 | ******* | strategy to explore test-drug(s) safety for pregnant women and their fetuses | 8 | 9 |
|  |  | 69 | ******* | participants who become pregnant are followed whether drug is discontinued or not | 8 | 9 |
|  | 5.2.assess the risk-benefit ratio by comparing the net risks of the research project with the potential benefits derived from collaborative partnership, social value, and respect for study populations | 70 |  | statement/discussion about risk-benefit ratio | 10 | 11 |
| 6.independent review | 6.1.ensure public accountability through reviews mandated by laws and regulations | 71 |  | reference to bioethics laws/regulations in host country or acknowledgement of absence thereof | 6 | 11 |
|  |  | 72 |  | reference to protocol review/approval process by ethics committee based in host community | 11 | 11 |
|  |  | 73 |  | acknowledgement of the right of regulatory body based in host community to discontinue trial | 9 | 11 |
|  | 6.2.ensure public accountability through transparency and reviews by other international and nongovernmental bodies, as appropriate | 74 |  | trial registered with an international trial registry | 11 | 11 |
|  |  | 75 |  | funding source(s)/ in-kind support disclosed | 11 | 11 |
|  |  | 76 |  | mentioned reference to/use of bioethics guidelines developed by host country | 2 | 11 |
|  |  | 77 |  | mention of international consultation or use of international guidelines | 9 | 11 |
|  | 6.3.ensure independence and competence of the reviews | 78 |  | specification of measures taken to ensure independence and competence of ethics review | 0 | 11 |
|  |  | 79 |  | specification of measures taken to prevent situations of conflict(s) of interest in ethics review | 0 | 11 |
|  |  | 80 | ***** | strategy to assist local ethics committee in reaching international standard procedures | 0 | 10 |
| 7.informed consent | 7.1.involve the community in establishing recruitment procedures and incentives | 81 |  | consultation with host community for appropriate recruitment procedures and compensation | 3 | 11 |
|  |  | 82 |  | recruitment method is explained and participants source(s) is/are specified | 10 | 11 |
|  | 7.2.disclose information in culturally and linguistically appropriate formats | 83 | ***** | information available to participants/community in local official language(s)/main dialect(s) | 10 | 10 |
|  |  | 84 |  | information disclosed in plain language and clearly stating potential risks for study participants | 10 | 11 |
|  |  | 85 |  | information disclosed to participants/community in a culturally sensitive manner | 9 | 11 |
|  | 7.3.implement supplementary community and familial consent procedures where culturally appropriate | 86 |  | discussion on relevance of supplementary community and familial consent procedures | 3 | 11 |
|  | 7.4.obtain consent in culturally and linguistically appropriate formats | 87 |  | informed consent is an iterative process | 10 | 11 |
|  |  | 88 |  | time allowed to read consent form off-study site before signing | 1 | 11 |
|  |  | 89 |  | specific modalities for conduct and documentation of consent process for illiterate participants | 8 | 11 |
|  |  | 90 |  | oral explanations given and opportunities to ask questions offered | 10 | 11 |
|  |  | 91 |  | systematic measures to assess comprehension of disclosed information by potential participant | 10 | 11 |
|  | 7.5.ensure the freedom to refuse or withdraw | 92 |  | description of measures to prevent/address coercion | 7 | 11 |
| 8.respect for recruited participants & study community | 8.1.develop and implement procedures to protect the confidentiality of recruited and enrolled participants | 93 |  | specification of procedures to protect confidentiality of recruited and enrolled participants | 11 | 11 |
|  | 8.2.ensure that participants know they can withdraw without penalty | 94 |  | systematic steps to insure that enrolled participants know their rights regarding withdrawing | 11 | 11 |
|  | 8.3.provide enrolled participants with information that arises in the course of the research study | 95 |  | statement about updating information disclosed to participants with relevant new information | 6 | 11 |
|  |  | 96 |  | consent to be confirmed in cases that may affect participant’s willingness to remain involved | 1 | 11 |
|  | 8.4.monitor and develop interventions for medical conditions, including research-related injuries, for enrolled participants at least as good as existing local norms | 97 |  | description of relevant local health care standards in each study site | 5 | 11 |
|  |  | 98 |  | description of health monitoring/care plan for study participants during trial period | 11 | 11 |
|  |  | 99 |  | discussion about appointing ombudsperson or partnering with independent organization | 1 | 11 |
|  |  | 100 |  | compensation plan for trial-related harm | 5 | 11 |
|  | 8.5.inform participants and the study community of the results of the research | 101 |  | statement about giving feedback to participants/host community about study results | 8 | 11 |
